# Levels, trends and correlates of unmet need for family planning among postpartum women in Indonesia: 2007–2015

**DOI:** 10.1186/s12905-017-0476-x

**Published:** 2017-11-28

**Authors:** Siswanto Agus Wilopo, Althaf Setyawan, Anggriyani Wahyu Pinandari, Titut Prihyugiarto, Flourisa Juliaan, Robert J. Magnani

**Affiliations:** 1 Department of Biostatistics, Epidemiology and Population Health and Center for Reproductive Health, Faculty of Medicine, Universitas Gadjah Mada, Gedung IKM Lantai 1, Jl Farmaco 1, Bulaksumur, Yogyakarta, 55281 Indonesia; 2 Center for Reproductive Health, Faculty of Medicine, Universitas Gadjah Mada, Yogyakarta, Indonesia; 3 National Population and Family Planning Bureau (BKKBN), Jakarta, Indonesia; 4 Avenir Health, 655 Winding Brook Drive, 4th floor, Glastonbury, 06033 CT USA

**Keywords:** Extended postpartum period, Contraceptive use, Demographic and health survey, PMA2020, Indonesia

## Abstract

**Background:**

Although Indonesia has relatively high contraceptive prevalence, postpartum family planning (PP-FP) has not been a particular point of emphasis. This article reports the results of analyses undertaken in order to (1) better understand levels and trends in unmet need for family planning among postpartum women, (2) assess the extent to which unmet need is concentrated among particular population sub-groups, and (3) assess the policy priority that PP-FP should have in relation to other interventions.

**Methods:**

The analyses were based on data from the 2007 and 2012 Indonesia Demographic and Health Surveys (IDHS) and the 2015 PMA2020 survey. Postpartum contraceptive use and unmet need were analyzed for fecund women who had given birth in the 3–5 years of preceding the respective surveys who were in the extended postpartum period at the time of the respective surveys. Factors associated with contraceptive use and unmet were assessed via multivariable logistic regressions using merged data from all three surveys. A wide range of biologic, demographic, socio-economic, geographic and programmatic factors were considered.

**Results:**

Contraceptive use during the extended postpartum period is high in Indonesia, with more than 74% of post-partum women reporting currently using a family planning method in the 2015 PMA2020 survey. This is up from 68% in 2007 and 70% in 2012. Total unmet need was 28% in 2007, falling slightly to 23% in 2012 and 24% in 2015. However, the timing of contraceptive initiation is less than optimal. By six months postpartum, only 50% of mothers had begun contraceptive use. Unmet need was highest among older women, women with 4+ children, with limited knowledge of contraceptive methods, making fewer ANC visits, from poor families and residents of islands other than Java and Bali.

**Conclusion:**

Unmet need for family planning among postpartum women in Indonesia is low in comparison with other low- and middle-income countries. However, because of limited durations of exclusive breastfeeding, many Indonesian women do not initiate contraception early enough after delivering children. Given already high contraceptive prevalence, targeting postpartum women for increased programmatic attention would seem strategically prudent.

## Background

After years of relative neglect, Postpartum Family Planning is currently receiving considerable global attention, most notably (but not exclusively) in connection with the global FP2020 initiative [1–4]. About one-quarter of inter-birth intervals in low- and middle-income countries are less than 24 months in length [5], thus exposing infants to risks of prematurity, low birthweight, and death, and exposing mothers to anemia, puerperal endometritis, premature rupture of membranes, and death [6, 7]. Inter-pregnancy intervals shorter than 18 months and longer than 59 months have been linked to increased risk of adverse perinatal outcomes [8]. Spacing pregnancies appropriately would help prevent such adverse perinatal outcomes [9].

From a programmatic view, period of postpartum is an appropriate time to provide birth planning education for women who delivered baby at the health care facility or had antenatal preceding child birth. Postpartum family planning interventions are premised on the assumptions that (1) demand for pregnancy prevention is particularly great following childbirth and (2) provision of services before discharge or at post-natal visits would be cost-effective. Postpartum family planning addresses the needs of those who wish to have children in the future or to space their pregnancy, as well as those who have reached their desired family size and wish to avoid future pregnancies or limit their number of children. Postpartum women are among those with the greatest unmet need for family planning. Yet they often do not receive the services they need to support longer birth intervals or reduce unintended pregnancy and its consequences. Research has shown that postpartum family planning can make significant contributions to efforts reduce unmet need, prevent unwanted pregnancies and increase birth spacing, all of which ultimately will increase maternal and child survival [10–13]. In view of this, it is entirely logical that countries seeking to more effectively help women and couples achieve their reproductive aspirations and improve maternal and child health outcomes would take a fresh look at postpartum family planning.

However, implementing postpartum family planning presents number of challenges. The period after delivery is challenging time during which a woman has to care for her newborn child as well as cope with cultural, emotional and physical changes. Beyond socio-cultural issues, insufficient integration of family planning with ante-natal care (ANC) services in many settings appears to be a formidable obstacle remaining to be overcome. There have been many successful and unsuccessful initiatives to integrate between family planning with maternal and child health services [7, 12–14]. Previous studies in Burkina Faso, Nepal, Senegal and Uganda indicate that even where family planning and ANC services are formally integrated, family planning topics are either rarely discussed during maternal and child health (MCH) consultations or are of little interest to postpartum women [15–17].

As in many other countries, Indonesia is engaging in the discussion regarding the need to strengthen postpartum family planning. In Indonesia, the need for universal access to reproductive health has been recognized as an essential global Sustainable Development Goal (SDG) target for 2030, an initiative to which the Government of Indonesia is committed [18]. On a more pragmatic level, the discussion about postpartum family planning in Indonesia is taking place in the context of assessing what can be done to revive stagnating growth in the modern of contraceptive prevalence rate (mCPR) and, as suggested by data from the most recent Performance Monitoring Accountability 2020 (PMA2020) survey in 2015, may be rising levels of unmet need for modern contraception compared to Indonesian Demographic Health Survey (IDHS) 2007 and 2012 [19–21]. Family planning in Indonesia is still undergoing a transition following government decentralization in the early 2000’s. Accompanying this transition has been a the emergence of significant differences in levels of government support for family planning across provinces and districts, as well as a significant rise in the private sector market share of family planning services and supplies. These developments have had significant implications for the manner in which family planning services are provided [22].

There is thus a clear need for further systematic study of postpartum family planning in the Indonesian context in order to (1) better understand levels and trends in unmet need among postpartum women, (2) assess the extent to which unmet need for postpartum family planning is concentrated among particular population sub-groups, and (3) assess the policy priority that postpartum family planning should have in relation to other interventions. This article reports the results of analyses undertaken to address the above points.

## Methods

The data used in the study consisted of samples of married women reporting recent births extracted from 2007 and 2012 Indonesia Demographic and Health Surveys (IDHS) and the 2015 Indonesia PMA2020 survey [19–21, 23]. Both the IDHS and the PMA2020 are nationally representative surveys of households and women of reproductive age (i.e., 15–49 years) based on stratified, multi-stage, cluster sample designs. All surveys were stratified by urban-rural, and the two IDHS by province as well. Household and individual female response rates were high in all surveys: 99% and 96% in the two IDHS, and 94% and 91% in the PM2020 survey.

Table 1 documents the numbers of sample households and married women of reproductive age selected in the respective surveys. Further analyses will be based on women who had delivered babies during the five (5) years before the two IDHS and in the three (3) years prior to PMA2020 data collection, and among these women that had not yet completed the extended postpartum period (i.e., 24 months) following the reference delivery at the time of the respective surveys. Background information on this group of women is also presented in the Table 1.

**Table 1:** Number of household, women age 15–49 interviewed, women giving births in the 3–5 years before the survey dates and sample of women considered postpartum

Sample Data	IDHS 2007		IDHS 2012		PMA-2020 2015		Total sample weighted	
	N	%	N	%	N	%	N	%
*Total households interviewed*								
Rural	24,477	60.1	22,986	52.4	5617	47.9	53,080	55.1
Urban	16,224	39.9	20,866	47.6	6109	52.1	43,199	44.9
Total	40,701	100.0	43,852	100.0	11,726	100.0	96,279	100.0
*Married women age 15–49 interviewed*								
Rural	12,662	41.5	16,224	49.2	3946	50.5	32,833	46.0
Urban	17,835	58.5	16,772	50.8	3870	49.5	38,476	54.0
Total	30,497	100.0	32,996	100.0	7816	100.0	71,309	100.0
*Married women giving birth in three years preceding survey*								
Rural	4006	41.5	4991	49.6	1095	51.5	10,091	46.2
Urban	5648	58.5	5072	50.4	1033	48.5	11,754	53.8
Total	9654	100.0	10,063	100.0	2128	100.0	21,845	100.0
*Sample Extended Postpartum (up to 24 months)*								
Rural	2869	41.9	3530	49.0	711	50.2	7111	46.0
Urban	3980	58.1	3678	51.0	704	49.8	8362	54.0
Total	6849	100.0	7208	100.0	1415	100.0	15,473	100.0
*Age Group*								
< 20	374	5.5	432	6.0	82	5.8	888	5.7
20–29	3665	53.5	3752	52.1	696	49.2	8113	52.4
≥ 30	2811	41.0	3024	41.9	638	45.1	6472	41.8
Total	6849	100.0	7208	100.0	1415	100.0	15,473	100.0
*Number of Children Even Born*								
1	2444	35.7	2780	38.6	491	34.7	5715	36.9
2	1947	28.4	2340	32.5	560	39.6	4847	31.3
3	1223	17.9	1148	15.9	225	15.9	2596	16.8
4+	1235	18.0	940	13.0	140	9.9	2314	15.0
Total								
*Knowledge of Contraceptive Method*								
< 4 methods	1296	18.9	1205	16.7	123	8.7	2624	17.0
≥ 4 methods	5546	81.1	6002	83.3	1292	91.3	12,840	83.0
Total	6842	100.0	7207	100.0	1415	100.0	15,464	100.0
*Place of Delivery* ^a^								
Home	3406	50	2207	30.6	–	–	5613	40
Institutional	3413	50	5007	69.4	–	–	8420	60
Total	6819	100	7214	100	–	–	14,033	100
*Antenatal care (ANC)* ^a^								
None	277	4.1	203	2.8	–	–	480	3.4
1	213	3.1	103	1.4	–	–	315	2.3
2–3	748	10.9	539	7.5	–	–	1286	9.2
4+	5596	81.9	6320	88.2	–	–	11,916	85.1
Total	6833	100	7165	100	–	–	13,998	100
*Visited by Family Planning Health Worker in the last 6 months*								
No	6445	94.2	6708	93.1	1261	89.1	14,413	93.2
Yes	400	5.8	494	6.9	154	10.9	1048	6.8
Total	6845	100	7201	100	1415	100	15,461	100
*Visited Health Care Facilities in the last 6 months*								
No	2924	42.7	2305	32.0	344	24.3	5574	36.0
Yes	3919	57.3	4899	68.0	1071	75.7	9889	64.0
Total	6844	100.0	7204	100.0	1415	100.0	15,463	100.0
*Sources of information from TV*								
No	4989	72.6	3801	52.6	621	44.2	9368	60.7
Yes	1884	27.4	3429	47.4	783	55.8	6070	39.3
Total	6874	100	7230	100	1404	100	15,438	100
*Sources of information from radio*								
No	6216	90.4	6574	90.9	1293	92.1	14,019	90.8
Yes	661	9.6	659	9.1	110	7.9	1424	9.2
Total	6878	100	7233	100	1403	100	15,443	100
*Sources of information from magazine or newspaper*								
No	6055	88	6187	85.5	1144	81.6	13,325	86.3
Yes	824	12	1049	14.5	258	18.4	2122	13.7
Total	6879	100	7236	100	1402	100	15,447	100
*Women Education Attainment*								
None	165	2.4	141	2.0	10	0.7	317	2.0
Primary	2645	38.6	2107	29.2	353	25.0	5105	33.0
Secondary	3447	50.3	4001	55.5	877	62.0	8325	53.8
Higher	592	8.6	958	13.3	175	12.3	1725	11.1
Total	6848	100.0	7208	100.0	1415	100.0	15,472	100.0
*Wealth Index*								
Poorest	1458	21.3	1482	20.6	217	15.4	3158	20.4
Poorer	1281	18.7	1504	20.9	251	17.8	3036	19.6
Middle	1406	20.5	1399	19.4	339	23.9	3143	20.3
Richer	1389	20.3	1461	20.3	300	21.2	3151	20.4
Richest	1315	19.2	1362	18.9	308	21.7	2985	19.3
Total	6849	100.0	7208	100.0	1415	100.0	15,473	100.0
*Residence*								
Urban	2869	41.9	3530	49.0	711	50.2	7111	46.0
Rural	3980	58.1	3678	51.0	704	49.8	8362	54.0
Total	6849	100.0	7208	100.0	1415	100.0	15,473	100.0
*Region*								
Java-Bali	3756	54.8	4006	55.6	863	61.0	8625	55.7
Other island	3093	45.2	3203	44.4	553	39.0	6848	44.3
Total	6849	100.0	7208	100.0	1415	100.0	15,473	100.0

^a^ *These variables are not available in the PMA2020 data*

The first dependent or outcome variable for the analysis, contraceptive use, was obtained from questions in the contraception section of the individual woman’s questionnaires of the respective surveys. Women were asked the question: Are you or your partner currently doing something or using any method to delay or avoid getting pregnant? If a woman reported that she was using any method, she was further queried as to type of method being used. Respondents were classified as being non-users, users of modern methods or users of traditional method on the basis of the answers to these questions.

The second dependent variable, postpartum unmet need, was constructed from 21 questions related to pregnancy and birth history, sexual activity, fertility preferences, and contraceptive use. This variable consists of two categories: spacing and limiting. The classic indicator of unmet need [24], updated by S Bradley, TN Croft, JD Fishel and CF Westoff [25], treats women as not in need of contraception as long as they remain amenorrhoeic for up to 24 months postpartum unless their last birth was unintended. This tends to underestimate unmet need for women whose last birth was intended but who want to avoid another pregnancy in the near future. In response, several revised “retrospective” and “prospective” definitions have been proposed, the relative merits of which are assessed in previous article [16] . We used the MR Borda, W Winfrey and C McKaig [26] definition of the “extended postpartum period - EPP” (i.e. 24 months) in the study. This definition will tend to overestimate unmet need in populations where exclusive breastfeeding is practiced for six months or more. In Indonesia, while a high proportion of newborns are ever breastfed (96%) [20], the duration of exclusive breastfeeding tends to be relatively short. Only 51% of births occurring in the five years prior to the 2012 IDHS were exclusively breastfed for one month, and only 3.4% were exclusively breastfed during months 6–7 was [20]. Thus, an unmet need definition that assumes limited protection from postpartum amenorrhea is sensible in the Indonesian context. Because of this limited protection provided by breastfeeding, the timing of initiating contraceptive use is important, and accordingly our analyses focus on contraceptive use and unmet need during four postpartum intervals: 2 months or less, at 6 months, at 12 months, and at 24 months.

In addition to assessing levels, trends and patterns of postpartum contraceptive use and unmet need, multivariable logistic regression techniques were used to assess the net associations of each of independent variable considered in the study with contraceptive use and unmet need during the extended postpartum period. The independent variables were selected for inclusion in the analysis based on their significance in previous studies of contraceptive behavior or on their hypothesized association with contraceptive use or unmet need for family planning. Our independent variables consisted of biologic, demographic, socio-economic, cultural, geographic and programmatic factors. The biologic and demographics variables are represented by age of women and number of their children (parity). Socio-economic variables comprise level of education and wealth index, while cultural variables relate to knowledge of contraceptive methods and their sources of information either from television, radio or magazine and newspaper. Women are also differentiated according to their residency (out site or within Java or Bali Islands) and urban-rural places. The programmatic variables cover number of antenatal care (ANC) frequency, place of delivery (home or at health institution), history of visiting health care facilities in the last 6 months, and visits by FP/health workers in the last 6 months. It should be noted that the data on place of delivery and ANC frequency are not available in the 2015 PMA2020 data. This incomparability of data will limit our modelling presented in this study.

Descriptive analyses were first undertaken to examine the distribution of possible determinants (explanatory variables) according to contraceptive uses and unmet need during extended postpartum period. Associations between the use of contraceptives and unmet need during the extended postpartum period on the one hand and the explanatory variables were assessed via simple logistic (bivariable associations) and multiple logistic regression (net associations). These associations were assessed based upon the size of odds ratios (OR) before and after adjustment. Best models between the independent variables were assessed by removing non-significant variables from the logistic models. The STATA version 15.0 software package was used to undertake the analyses [27].

Analyses were initially undertaken for each the respective data set, and then of a combined, weighted data set consisting of the data from all three surveys. Standardized weights were applied based upon the sample weights in the respective data sets [19–21]. In the multivariable analyses of the merged data set, we included a variable for “survey round” to control for possible unobserved secular and/or survey-specific influences.

## Results

Respondent background characteristics by survey year and in the merged data set were provided in Table 1. In the merged data set, a small majority of respondents lived in rural areas (54%) and on the islands of Java and Bali (56%). A plurality of respondents was 20–29 years of age and had borne either one or two children. The median education level was secondary school. About two-thirds had visited a health facility in the prior six months, 60% the reference births for the analyses were delivered at a health facility, and 85% of births were preceded by four or more ANC service visits. Well over 80% of respondents were aware of four or more contraceptive methods.

It will be noted, however, that the merged data set masks important secular trends in some of these characteristics. Most notable among these are increases in the proportions of women who had visited health facilities on the previous six months, delivered at a health facility, made four-plus ANC visits, were aware of four or more contraceptive methods, and had attained secondary levels of education or higher. Increased proportions of respondents living in urban areas and on the islands of Java and Bali are also evident. The proportion of women with four or more live births appears to be in steady decline.

**Fig. 1:**
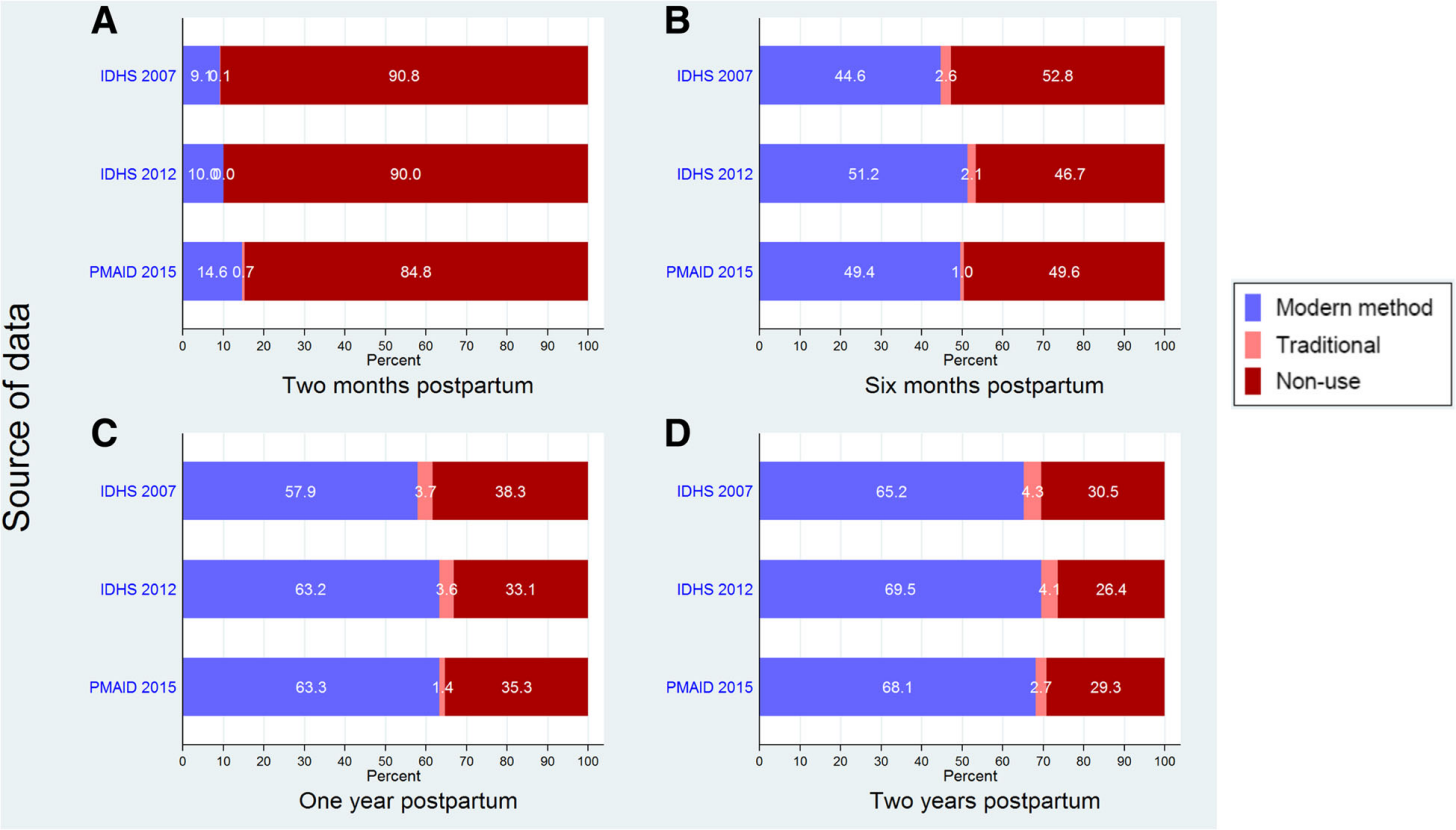
Type of contraceptive method used for during <2 months (**a**), <6 months (**b**), <12 months (**c**) and <24 months (**d**) postpartum according to IDHS 2007, IDHS 2012 and PMA 2015

Figure 1 displays data on contraceptive use during the extended postpartum period by number of months since delivery (less than 2, 6, 12 and 24 months). Prevalence of contraceptive use during the postpartum period is high in Indonesia, with 68% of women reporting using a modern family planning method by the end of the extended postpartum period in the 2015 PMA2020 survey (Fig. 1-D). This is up from 65% in 2007, but slightly lower than the 69.5% estimate from the 2012 IDHS. It is noteworthy that this figure is higher than the 2015 PMA2020 mCPR estimate for all married women (60%). Traditional method use, including abstinence, was low in all surveys.

Adoption of contraception in the first two months postpartum is relatively infrequent (about 15% in 2015), although the 2015 PMA2020 survey data suggest that this may be increasing, perhaps indicating an uptick in postpartum family planning. Modern contraceptive use rises to around 50% by six months postpartum in the 2012 and 2015 data and 63% by 12 months postpartum in both surveys.

**Fig. 2:**
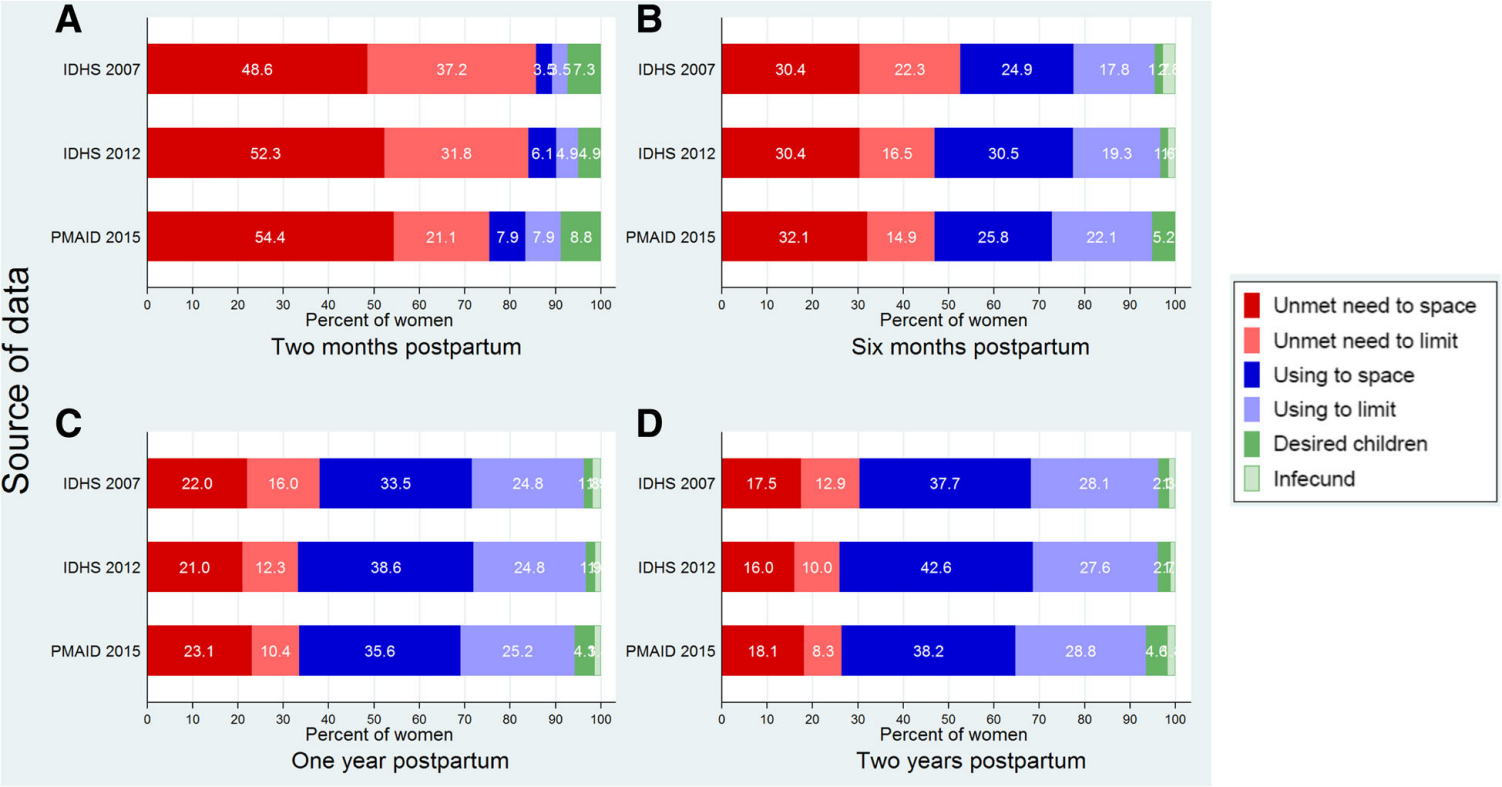
Meet need and unmet need during <2 months (**a**), <6 months (**b**), <12 months (**c**) and <24 months (**d**) postpartum according to IDHS 2007, IDHS 2012 and PMAID 2015

Survey results concerning levels of unmet need for family planning during the extended postpartum period are shown in Fig. 2. Total unmet need (spacing plus limiting) for the full extended postpartum period was 30.4% in 2007. This number declined to 26.0% in 2012, increasing slightly to 26.4% in 2015. In the first two months postpartum, unmet was 85.8% in 2007, falling slightly to 84.1% in 2012 and 75.5% in 2015 (Fig. 2-A). The decline is entirely accounted for by a reduction in the level of unmet need for limiting (from 37.2% in 2007 to 21.1% in 2015). In postpartum months 0–24 (Fig. 2-D), unmet need was 30.4% in 2007, falling to about 26.0% in both 2012 and 2015, again accounted for by a decline in unmet for limiting (from 12.9% in 2007 to 8.3% in 2015). Unmet need for spacing was consistently higher than unmet need for limiting. The level of unmet declines steadily over the postpartum period and as contraception is adopted, with no apparent trend over time except during the first two months postpartum, where unmet need appears to be on the rise.

**Fig. 3:**
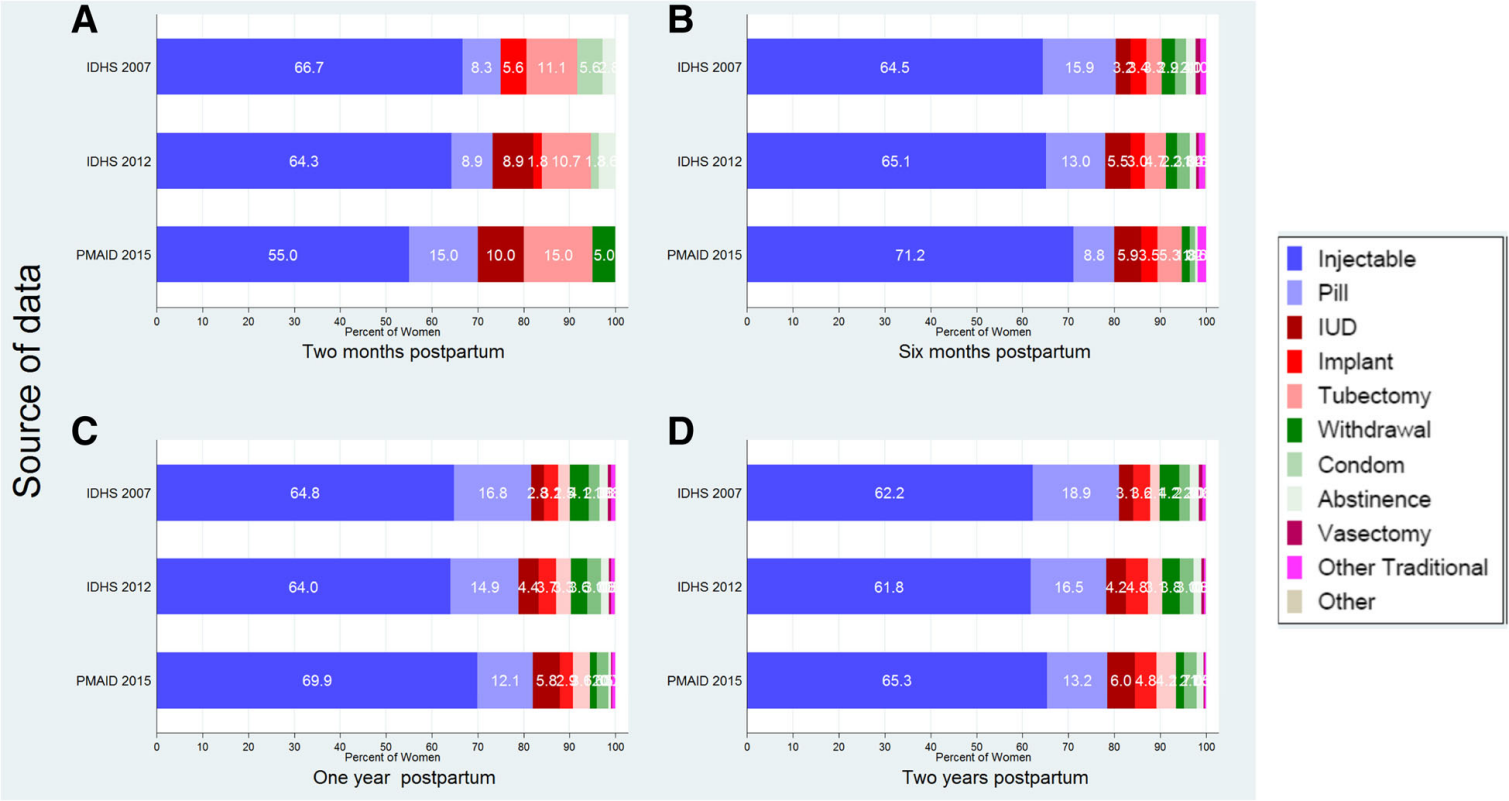
Mixed Contraception used during <2 months (**a**), <6 months (**b**), <12 months (**c**) and <24 months (**d**) postpartum according to IDHS 2007, IDHS 2012 and PMAID 2015

With regard to contraceptive method mix among contraceptive adopters during the extended postpartum period, injectable was by far the most popular method, accounting for over 60% of the method mix in all three surveys (Fig. 3). Oral contraceptives were the second most popular method at 13–19% depending upon survey, with a declining trend except for during the first two months postpartum. These two methods jointly account for almost 80% of the postpartum method mix in all three surveys. The skewed postpartum method mix favoring short-term methods mimics that among all contraceptive users in Indonesia [18–20]. Male family planning users (condom use and vasectomy) remain infrequent. Formal use of the lactational amenorrhea method (LAM), withdrawal, abstinence and other traditional methods are rare in Indonesia. It should be noted that tubectomy and IUD use are as share of method mix the most common in the less than two months postpartum period, perhaps reflecting recent efforts to promote longer-term methods as part of postpartum family planning efforts.

In order to examine factors associated with postpartum use of contraceptives and unmet need for family planning, bivariate and multivariable logistic regressions were undertaken. As described in the Methodology section of this article, analyses were undertaken of each survey data set individually and then of a merged data set. As the analyses of the separate survey data files and the merged data set yielded similar and consistent results, only the results of the analyses of the merged data set are presented here. The results after the removal of factors that were not associated with postpartum contraceptive use and unmet need in bivariate analyses are shown in Tables 2 and 3, respectively. Both crude and adjusted odds-ratios (ORs), along with 95% confidence intervals for both, are shown in the tables.

**Table 2:** Unadjusted (model 1) and adjusted (model 2--4) odds ratios determinants of use of modern contraceptives among women at extended postpartum total sample

	Model 1	Model 2^++^	Model 3	Model 4
Modern Contraceptive Use				
*Age Group*				
< 20	1 (1,1)	1 (1,1)	1 (1,1)	1 (1,1)
20–29	0.92 (0.79,1.06)	0.79^**^ (0.67,0.94)	0.80^**^ (0.68,0.93)	0.80^**^ (0.68,0.93)
> =30	0.73^***^ (0.63,0.85)	0.62^***^ (0.52,0.75)	0.65^***^ (0.55,0.78)	0.65^***^ (0.55,0.78)
*Number of Children Even Born*				
1	1 (1,1)	1 (1,1)	1 (1,1)	1 (1,1)
2	1.28^***^ (1.18,1.39)	1.40^***^ (1.27,1.55)	1.38^***^ (1.26,1.51)	1.38^***^ (1.26,1.51)
3	1.07 (0.97,1.18)	1.29^***^ (1.14,1.46)	1.26^***^ (1.12,1.41)	1.26^***^ (1.12,1.41)
4+	0.65^***^ (0.59,0.71)	0.94 (0.82,1.07)	0.87^*^ (0.77,0.99)	0.87^*^ (0.77,0.98)
*Knowledge of Contraceptive Method*				
< 4 methods	1 (1,1)	1 (1,1)	1 (1,1)	1 (1,1)
> =4 methods	1.78^***^ (1.65,1.93)	1.41^***^ (1.28,1.56)	1.53^***^ (1.40,1.68)	1.54^***^ (1.41,1.69)
*Place of Delivery* ^*$*^				
Non- institutional (home)	1 (1,1)	1 (1,1)	–	–
Institutional	1.28^***^ (1.19,1.37)	0.92 (0.84,1.01)		
*Antenatal care (ANC)* ^*$*^				
None	1 (1,1)	1 (1,1)	–	–
1	1.83^***^ (1.42,2.36)	1.51^**^ (1.16,1.96)		
2–3	2.39^***^ (1.99,2.86)	1.92^***^ (1.59,2.33)		
4+	3.85^***^ (3.29,4.51)	2.77^***^ (2.33,3.30)		
*Visited by Family Planning Health Worker in the last 6 months*				
No	1 (1,1)	1 (1,1)	1 (1,1)	–
Yes	1.06 (0.93,1.21)	1.05 (0.90,1.21)	1.06 (0.93,1.22)	
*Visited Health Care Facilities in the last 6 months*				
No	1 (1,1)	1 (1,1)	1 (1,1)	–
Yes	1.23^***^ (1.15,1.31)	1.04 (0.96,1.12)	1.09^*^ (1.01,1.17)	
*Sources of information from TV*				
No	1 (1,1)	1 (1,1)	1 (1,1)	1 (1,1)
Yes	1.29^***^ (1.20,1.38)	1.09 (1.00,1.19)	1.14^**^ (1.05,1.24)	1.13^**^ (1.05,1.23)
*Sources of information from radio*				
No	1 (1,1)	1 (1,1)	1 (1,1)	–
Yes	0.95 (0.85,1.07)	0.93 (0.81,1.06)	0.90 (0.80,1.02)	
*Sources of information from magazine or newspaper*				
No	1 (1,1)	1 (1,1)	1 (1,1)	1 (1,1)
Yes	0.90^*^ (0.82,0.99)	0.82^***^ (0.73,0.92)	0.81^***^ (0.73,0.91)	0.81^***^ (0.72,0.90)
*Women Education Attainment*				
No education	1 (1,1)	1 (1,1)	1 (1,1)	1 (1,1)
Primary	2.84^***^ (2.32,3.46)	1.55^***^ (1.24,1.93)	1.96^***^ (1.59,2.41)	1.98^***^ (1.61,2.44)
Secondary	3.32^***^ (2.73,4.05)	1.40^**^ (1.11,1.75)	1.77^***^ (1.43,2.19)	1.79^***^ (1.45,2.22)
Higher	2.19^***^ (1.77,2.70)	0.93 (0.72,1.20)	1.13 (0.89,1.43)	1.14 (0.90,1.45)
*Wealth Index*				
Poorest	1 (1,1)	1 (1,1)	1 (1,1)	1 (1,1)
Poorer	1.74^***^ (1.58,1.91)	1.45^***^ (1.30,1.62)	1.52^***^ (1.38,1.69)	1.53^***^ (1.38,1.70)
Middle	1.85^***^ (1.67,2.04)	1.46^***^ (1.29,1.65)	1.57^***^ (1.40,1.76)	1.57^***^ (1.40,1.76)
Richer	1.78^***^ (1.61,1.96)	1.44^***^ (1.26,1.65)	1.54^***^ (1.36,1.74)	1.55^***^ (1.37,1.74)
Richest	1.48^***^ (1.33,1.63)	1.32^***^ (1.14,1.54)	1.38^***^ (1.21,1.59)	1.39^***^ (1.21,1.59)
*Residence*				
Urban	1 (1,1)	1 (1,1)	1 (1,1)	1 (1,1)
Rural	0.87^***^ (0.81,0.93)	1.12^**^ (1.03,1.23)	1.10^*^ (1.02,1.19)	1.11^*^ (1.02,1.20)
*Region*				
Other Islands	1 (1,1)	1 (1,1)	1 (1,1)	1 (1,1)
Java-Bali	1.58^***^ (1.46,1.71)	1.33^***^ (1.21,1.46)	1.37^***^ (1.25,1.49)	1.38^***^ (1.26,1.50)
*Source of Data*				
IDHS 2007	1 (1,1)	1 (1,1)	1 (1,1)	1 (1,1)
IDHS 2012	1.22^***^ (1.14,1.30)	1.21^***^ (1.12,1.30)	1.22^***^ (1.13,1.31)	1.23^***^ (1.14,1.32)
PMA 2015	1.19^**^ (1.06,1.35)	–	0.96 (0.85,1.10)	0.99 (0.87,1.12)
Pseudo *R* ^2^		0.046	0.038	0.038
AIC		17,345.1	19,453.7	19,485.2
df_m		25	22	19
Observations	15,414^***$***^	13,857	15,415	15,433

^++^Model 2 excluded data PMA 2015 so the sample size smaller than other models

Exponentiated coefficients; 95% confidence intervals in brackets

Likelihood Ratio (LR) from Akaiki (AIC),

df_m = Degrees of freedom of the model,

^$^Since the PMA 2015 survey did not collect information on place of delivery or receipt of ANC services, the sample size for these variables was only 13,944

^*^ *p* < 0.05

^**^ *p* < 0.01

^***^ *p* < 0.001

**Table 3:** Unadjusted (model 1) and adjusted (model 2--4) odds ratios determinants of unmet need of family planning among women at extended postpartum total sample

	Model 1	Model 2	Model 3	Model 4
Unmet Need for Modern Contraceptive Use				
*Age Group*				
< 20	1 (1,1)	1 (1,1)	1 (1,1)	–
20–29	1.00 (0.86,1.17)	1.13 (0.95,1.34)	1.12 (0.95,1.31)	–
> =30	1.18^*^ (1.01,1.38)	1.24^*^ (1.02,1.51)	1.18 (0.99,1.42)	–
*Number of Children Even Born*				
1	1 (1,1)	1 (1,1)	1 (1,1)	1 (1,1)
2	0.84^***^ (0.77,0.92)	0.80^***^ (0.72,0.89)	0.82^***^ (0.75,0.91)	0.85^***^ 0.77,0.93)
3	1.03 (0.93,1.14)	0.92 (0.81,1.05)	0.96 (0.85,1.09)	1.01 (0.91,1.13)
4+	1.63^***^ (1.48,1.80)	1.25^**^ (1.09,1.43)	1.35^***^ (1.19,1.54)	1.45^***^ (1.31,1.60)
*Knowledge of Contraceptive Method*				
< 4 methods	1 (1,1)	1 (1,1)	1 (1,1)	1 (1,1)
> =4 methods	0.54^***^ (0.50,0.59)	0.72^***^ (0.65,0.80)	0.66^***^ (0.60,0.72)	0.66^***^ (0.60,0.73)
*Place of Delivery*				
Non- institutional	1 (1,1)	1 (1,1)	–	–
Institutional	0.68*** (0.63,0.73)	0.97 (0.89,1.07)		
*Antenatal care ANC)*				
None	1 (1,1)	1 (1,1)	–	–
1	0.66^**^ (0.52,0.85)	0.75^*^ (0.58,0.98)		
2–3	0.51^***^ (0.42,0.60)	0.60^***^ (0.50,0.73)		
4+	0.30^***^ (0.25,0.34)	0.42^***^ (0.36,0.50)		
*Visited by Family Planning Health Worker in the last 6 months*				
No	1 (1,1)	1 (1,1)	1 (1,1)	–
Yes	0.96 (0.84,1.11)	1.01 (0.86,1.18)	0.96 (0.83,1.10)	
*Visited Health Care Facilities in the last 6 months*				
No	1 (1,1)	1 (1,1)	1 (1,1)	–
Yes	0.85^***^ (0.79,0.91)	1.02 (0.94,1.11)	0.97 (0.90,1.05)	
*Sources of information from TV*				
No	1 (1,1)	1 (1,1)	1 (1,1)	1 (1,1)
Yes	0.76^***^ (0.70,0.82)	0.92 (0.84,1.02)	0.91^*^ (0.83,0.99)	0.91^*^ (0.84,0.99)
*Sources of information from radio*				
No	1 (1,1)	1 (1,1)	1 (1,1)	–
Yes	0.96 (0.85,1.08)	1.04 (0.90,1.19)	1.04 (0.91,1.19)	
*Sources of information from magazine or newspaper*				
No	1 (1,1)	1 (1,1)	1 (1,1)	1 (1,1)
Yes	0.99 (0.89,1.09)	1.19^**^ (1.04,1.35)	1.21^**^ (1.07,1.36)	1.21^**^ (1.08,1.36)
*Women Education Attainment*				
No education	1 (1,1)	1 (1,1)	1 (1,1)	1 (1,1)
Primary	0.49^***^ (0.40,0.59)	0.88 (0.71,1.10)	0.70^***^ (0.57,0.86)	0.70^***^ (0.57,0.85)
Secondary	0.38^***^ (0.31,0.46)	0.91 (0.73,1.14)	0.73^**^ (0.59,0.90)	0.73^**^ (0.59,0.90)
Higher	0.46^***^ (0.37,0.57)	1.20 (0.92,1.56)	0.99 (0.78,1.26)	1.01 (0.79,1.28)
*Wealth Index*				
Poorest	1 (1,1)	1 (1,1)	1 (1,1)	1 (1,1)
Poorer	0.61^***^ (0.55,0.68)	0.75^***^ (0.67,0.84)	0.71^***^ (0.63,0.78)	0.71^***^ (0.64,0.79)
Middle	0.54^***^ (0.49,0.60)	0.72^***^ (0.63,0.82)	0.66^***^ (0.58,0.74)	0.67^***^ (0.60,0.75)
Richer	0.54^***^ (0.48,0.60)	0.71^***^ (0.62,0.82)	0.65^***^ (0.57,0.74)	0.67^***^ (0.59,0.76)
Richest	0.57^***^ (0.51,0.64)	0.71^***^ (0.60,0.84)	0.66^***^ (0.57,0.77)	0.69^***^ (0.60,0.79)
*Residence*				
Urban	1 (1,1)	1 (1,1)	1 (1,1)	–
Rural	1.27^***^ (1.18,1.36)	0.93 (0.85,1.03)	0.95 (0.87,1.04)	
*Region*				
Other Islands	1 (1,1)	1 (1,1)	1 (1,1)	1 (1,1)
Java-Bali	0.68^***^ (0.62,0.74)	0.87^**^ (0.79,0.97)	0.83^***^ (0.76,0.91)	0.83^***^ (0.76,0.91)
*Source of Data*				
IDHS 2007	1 (1,1)	1 (1,1)	1 (1,1)	1 (1,1)
IDHS 2012	0.81^***^ (0.75,0.87)	0.84^***^ (0.78,0.91)	0.82^***^ (0.76,0.88)	0.82^***^ (0.76,0.88)
PMA 2015	0.84^**^ (0.73,0.95)		1.03 (0.90,1.18)	1.02 (0.89,1.17)
Pseudo *R* ^2^		0.038	0.029	0.029
AIC		15,872.6	17,787.3	17,808.5
df_m		25	22	16
Observations		13,857	15,415	15,433

Exponentiated coefficients; 95% confidence intervals in brackets

Likelihood Ratio (LR) from Akaiki, (AIC)

df_m = Degrees of freedom of the model,

^ Include PMA2020

^Not Include due to missing information in PMA2020

^*^ *p* < 0.05

^**^ *p* < 0.01

^***^ *p* < 0.001

Bivariate logistics regressions (Table 2, Model 1), which include the full set of explanatory factors considered in the analyses, reveal that a sizeable number of factors were significantly associated with contraceptive use during the postpartum period. These include age, children ever born, knowledge of contraceptive use (four methods or more), place of delivery, antenatal care for the most recent birth (ANC), visited health facilities in the last 6 months, source of information on contraception from several media outlets (television, magazines and newspapers, but not radio), educational attainment, wealth status, urban vs. rural residence and region (Java - Bali vs. other islands).

Factors that achieved statistical significance in the bivariate analyses were retained in the multiple logistic regressions, the results of the first of which are shown in Table 2, Model 2. Note that this model excludes PMA2020 2015 survey data since that survey did not measure place of delivery or antenatal care for the last birth. Three factors that were significant on the bivariate analyses drop out when the effects of other factors considered in the analyses are taken into account – place of delivery (facility vs. home), having visited a health facility in the last six months, and TV as a source of information on family planning. The lack of net association of place of delivery with postpartum contraceptive use is likely the result of its association with other variables under consideration (e.g., urban-rural residence, region, family wealth status, and perhaps receipt of ANC services), as well as the heretofore insufficient priority assigned to postpartum family planning in Indonesia. Among the four health services-related variables considered in the analyses, only number of ANC service visits made prior to the delivery of the reference birth remained significant when the effects of other factors were controlled. The association of postpartum family planning with number ANC visits suggests a dose-response relationship (at least up to four visits), with increasingly large adjusted ORs of having adopted a contraceptive method during the extended postpartum period with increasing number of ANC visits.

The other factors with the strongest net associations with postpartum contraceptive adoption were women’s age, number of children ever born (i.e., including the reference child), knowledge of four or more contraceptive methods, educational attainment, family wealth and residence on the islands of Java or Bali. Women in the both the 20–29 and 30+ age groups less likely to use contraceptives than women less than 20 years of age (AORs = 0.79 and 0.62, respectively). The results also indicate that the number of children ever born influence postpartum use of contraception. Women with 2–3 children are 1.3–1.4 times more likely to use contraceptives than women with one child, while women with four or more children were no more likely to have adopted contraception in the extended postpartum period than women with one child.

Knowledge of four or more contraceptives methods was strongly associated with contraceptive use (AOR = 1.53) after adjustment for other variables. Not surprisingly, education and family wealth were also strongly associated with postpartum use of contraceptives, but the pattern of differences within categories of these variables merits attention. With regard to education, women with primary and secondary education were about 1.5 times more likely to have used contraception than women with no education. However, women with above secondary-level education were indistinguishable from women with no education with regard to likelihood of having adopted a contraceptive method. For the family wealth, women in the four highest wealth quintiles were 1.3 to 1.5 times as likely to have adopted a contraceptive method by the end of the extended postpartum period as women in the lowest quintile. However, no gradient or dose-response effect is observed across the four quintiles – women in all four wealth quintiles had about the same likelihood of having adopted a method. It is only women in the lowest wealth quintile that stand out in terms of probability of adopting a contraceptive method in the extended postpartum period.

Women living on the islands of Java and Bali were one-third more likely to have used contraceptives in comparison with residents of other islands. This likely reflects inter-island differences in demand for family planning and supply-side readiness to provide services. When the other factors considered in the analyses are controlled statistically, women residing in rural were about 12% more likely to have adopted a contraceptive method postpartum than residents of urban areas. This is a testament to the reach of the Indonesian national family planning program. However, it also indicates the need to better reach the urban poor with postpartum family planning services.

In the Model 3 - Table 2, we excluded the place of delivery and antenatal care variables as these variables were not measured in the 2015 PMA2020 survey. All other variables in the model were measured similarly in the PMA2020 survey as in the 2007 and 2012 IDHS. With regard to results, other than slightly stronger associations of receipt of family planning of information from television and education, the results of Model 3 are essentially the same as for Model 2. In Model 4 - Table 2, only factors that were statistically significant in Model 3 were retained. The results are for all intents and purposes identical to those in Model 3.

Comparable multivariable analyses were undertaken of factors associated with unmet need for family planning among postpartum women during the period 2007–2015, the results of which are displayed in Table 3. The results point to same set of factors as were observed as being strongly associated with postpartum contraceptive use as being associated with unmet need. Higher unmet need is associated with higher age, higher parity, lower knowledge of family planning methods, fewer ANC visits, non-receipt of family planning information from TV, receipt of family planning information from magazines or newspaper, no formal education, lowest quintile family wealth, and residence on islands other than Java or Bali.

## Discussion

Global estimates of the level of unmet need among postpartum women vary from 32% to 62%, depending upon the definition used [16]. While these estimates vary with regard to the countries included and time reference, it is nevertheless apparent that at 26.4% unmet need among postpartum women in Indonesia (weighted average 2007–2015) is lower than that found in many other low- and middle-income countries. Insofar as the modern contraceptive prevalence rate in Indonesia has exceeded 50% since the early 2000s, this perhaps should not come as a surprise [19].

That being said, unmet need among postpartum women in Indonesia is substantially higher than among married women of reproductive age in general (11.4% in the 2012 IDHS and 15.3% on the 2015 PMA2020 survey), and on this basis is worthy of programmatic attention [19, 20]. Although Indonesia had early success with family planning in comparison with the other low- and middle-income countries, the mCPR has stagnated in recent years [19–21], largely the result of the transition to a decentralized system of government beginning in the early 2000s that remains a work in progress [28]. Countries with mCPRs of 60% or higher such as Indonesia have already provided information on and access to family planning services to a high proportion of women and couples of reproductive ages, and as such the primary focus should be on reaching remaining under-served sub-populations and improving service quality to, among other things, reduce the relatively high rate of method discontinuation still found in the country. In this context, addressing postpartum unmet need would be make great strategic sense in order to make the health system more responsive to the reproductive needs and intentions of Indonesian women and families.

Although postpartum contraceptive use in Indonesia is relatively high, the timing of initiation is less than optimal. For example, in 2013 60% or more of Indonesian mothers had discontinued exclusive breastfeeding by six months postpartum [29], but only 50% had begun contraceptive use. Although the Indonesian Ministry of Health aspires to at least double the percent of mothers who exclusively breastfeed for six months, achieving this goal will take time, and even if achieved the six-month exclusive breastfeeding rate will remain low by international standards. From this perspective as well, prioritizing postpartum family planning makes strategic sense.

Another issue for concern is the highly-skewed method mix among postpartum contraceptive users [18]. The contraceptive method mix among all users in Indonesia is skewed toward short-term methods – injections and oral contraceptives in particular, with 75% of all users in the 2015 PMA2020 survey reporting using these two methods. The method mix among postpartum contraceptive users is even more skewed – over 80% using injections or orals [21]. In view of the fact that about 10% of total demand for family planning among postpartum women in the aggregate during the 2007–2015 period was for limiting, there would appear to have been many missed opportunities to engage women who desire no more children in the use of longer-acting reversible contraceptives (LARCs). Promoting LARCs among such women would be consistent with the Government of Indonesia’s Medium-Term Development Plan 2015–2019 (RPJMN) [18], which seeks to increase the market share of LARCs among contraceptive users from 14% to 23%.

The results of the multivariable analyses concerning differentials by background characteristics also raise some concerns. Two differentials in particular merit programmatic attention. First, the substantially higher level of unmet need observed among residents of islands other than Java and Bali points to a need to improve the reach and quality of family planning services, and health services in general, on these outer islands. This is also the case with regard to low income families nationwide. The magnitude of household wealth differentials in contraceptive during the extended postpartum period appear to be larger than those among all married women, suggesting that the services being provided to women from the poorest households may be neglecting postpartum family planning to larger extent than those provided to other women. This is likely associated with differences in place of delivery and use of ANC and postpartum maternal health services. In principle, the inclusion of most family planning services in the new universal social health insurance scheme (Jaminan Kesehatan Nasional - JKN) scheduled for full national coverage by 2019 should help alleviate this concern, but many challenges remain in rolling out the scheme to achieve universal coverage [30].

As noted by IH Shah, KG Santhya and J Cleland [1], the ideal strategy for improving family planning program performance is to incorporate contraceptive advice and services across the continuum of reproductive health care. The steady increase in the proportion of births delivered in health facility more or less across the globe expands the number of opportunities for such a comprehensive approach. However, as IH Shah, KG Santhya and J Cleland [1] also correctly observe, given competing priorities and pressure on budgets and staff, policy and program choices have to be made, choices that are often constrained by the policy, programmatic, and cultural contexts. More specifically, effective implementation requires more effective integration of family planning with other services accessed during pregnancy and the postpartum period than currently exists in many countries, Indonesia included. Hopefully, however, this recognition will galvanize policies and programmatic action with regard to universal access to reproductive health services for all in Indonesia in relation to national SDG goals.

## Conclusions

Unmet need for family planning among postpartum women in Indonesia is low in comparison with other low- and middle-income countries. However, because of limited durations of exclusive breastfeeding, many Indonesian women do not initiate contraception early enough after delivering children, thus exposing themselves to the risk of unplanned pregnancies. These risks are concentrated among older women, women with 4+ children, women with limited knowledge of contraceptive methods, women making fewer ANC visits, women from poor families and residents of islands other than Java and Bali. Given already high contraceptive prevalence, targeting postpartum women for increased programmatic attention, particularly with regard to the use of LARCs for women desiring not to have any further children, would seem prudent strategically.

## Abbreviations

ANC: Antenatal Care
AOR: Adjusted Odds Ratio
BMGF: Bill and Melinda Gates Foundation
EPP: Extended Postpartum Period
FP: Family Planning
FP2020: Family Planning 2020
IDHS: Indonesia Demographic Health Survey
IUD: Intra Uterine Device
JHU: John Hopkins University
JKN: National Health Insurance
LAM: Lactational Amenorrhea Method
LARC: Longer Acting Reversible Methods
MCH: Maternal and Child Health
mCPR: Modern Contraceptive Prevalence Rate
OR: Odds Ratio
PMA2020: Performance Monitoring and Accountability 2020
PP-FP: Postpartum Family Planning
RPJMN: *Rencana Pembangunan Jangka Menegah Nasional* (Medium Term Development Plan)
SDGs: Sustainable Development Goals
